# 92. Obeldesivir Reduced SARS-CoV-2 Infectious Titers in the BIRCH Phase 3 Clinical Trial

**DOI:** 10.1093/ofid/ofae631.029

**Published:** 2025-01-29

**Authors:** Lauren Rodriguez, Anca Streinu-Cercel, Juan M Gonzalez Del Castillo, Yiannis Koullias, Afsaneh Mozaffarian, Kim Etchevers, Shuguang Chen, Robert H Hyland, Joe Llewellyn, Romas Geleziunas, Leon Fouche, Shan-Chwen Chang, Antonella Castagna, Charlotte Hedskog

**Affiliations:** Gilead Sciences, Inc., Foster City, CA; "Prof. Dr. Matei Bals" National Institute for Infectious Diseases Carol Davila Medicine and Pharmacy University, Bucharest, Bucuresti, Romania; Emergency Department, Hospital Clínico Universitario San Carlos, Madrid, Madrid, Spain; Gilead Sciences, Inc., Foster City, CA; Gilead Sciences, Inc., Foster City, CA; Gilead Sciences, Inc., Foster City, CA; Gilead Sciences, Inc, Foster City, California; Gilead Sciences, Inc., Foster City, CA; Gilead Sciences, Inc., Foster City, CA; Gilead Sciences Inc., Foster City, California; Limpopo Clinical Research Initiative, Thabazimbi, Limpopo, South Africa; National Taiwan University Hospital, Taipei, Taipei, Taiwan (Republic of China); IRCCS San Raffaele Hospital and Vita-Salute San Raffaele University, Milano, Lombardia, Italy; Gilead Sciences, Inc., Foster City, CA

## Abstract

**Background:**

COVID-19 remains a serious condition, especially for those with certain risk factors. Obeldesivir (ODV) is an oral, broad spectrum, nucleoside analog prodrug inhibitor of SARS-CoV-2 RNA-dependent RNA polymerase.

**Methods:**

BIRCH was a Phase 3, randomized, double-blind, placebo-controlled trial to evaluate the efficacy and safety of a 5-day ODV treatment in adults with risk factors for developing severe COVID-19. Nasal swab samples were collected on Days 1, 3, 5, 10, and 15. For participants with baseline viral load (VL) ≥10^6^ copies/mL, samples were evaluated for SARS-CoV-2 infectious titers. In participants with baseline infectious titer ≥200 plaque forming units (PFU)/mL, Day 3 or 5, and, if available, Day 10 samples were assessed. Infectious titer assays were performed by inoculating Vero E6 cells with nasal swab sample serial dilutions, incubating for 96 hours with a carboxymethylcellulose overlay, and quantifying PFU following staining of the cell monolayer. The proportion of participants with undetectable infectious titers was compared between the ODV and placebo (PBO) groups using Fisher’s exact test. Additionally, change from baseline in infectious titer was compared between the ODV and PBO groups using a mixed model repeated measures approach.

**Results:**

The proportion of participants with positive infectious titers at baseline (≥200 PFU/mL) was similar between the ODV (74/137 [54%]) and PBO (72/129 [56%]) groups. ODV treatment reduced VL at Day 5 versus PBO (–0.58 log_10_ copies/mL; *P* < 0.0001) and led to significantly greater reductions from baseline in infectious titers on Day 3 (−0.54 log_10_ PFU/mL; *P* = 0.0003) and Day 5 (−0.17 log_10_ PFU/mL; *P* = 0.0014) compared to PBO. At Day 3, 35/44 participants (80%) in the ODV group and 18/37 participants (49%) in the PBO group were negative for infectious virus (*P* = 0.0049). At Day 5, 68/68 participants (100%) in the ODV group and 56/69 participants (81%) in the PBO group were negative for infectious virus (*P* = 0.0001). At Day 10, the proportions of participants negative for infectious virus were similar between the groups (ODV: 47/49 [96%]; PBO: 40/41 [98%]: *P* = 1.000).

**Conclusion:**

In BIRCH, ODV reduced SARS-CoV-2 infectious titers and VL, demonstrating inhibition of SARS-CoV-2 replication in adults with risk factors for developing severe COVID-19.

**Disclosures:**

**Lauren Rodriguez, PhD**, Gilead Sciences, Inc.: Employee|Gilead Sciences, Inc.: Stocks/Bonds (Public Company) **Anca Streinu-Cercel, MD PhD Prof. Infectious Diseases**, Gilead Sciences, Inc.: Grant/Research Support|Gilead Sciences, Inc.: Honoraria **Yiannis Koullias, MD**, Gilead Sciences, Inc.: Employee|Gilead Sciences, Inc.: Stocks/Bonds (Public Company) **Afsaneh Mozaffarian, MS**, Gilead Sciences, Inc.: Employee|Gilead Sciences, Inc.: Stocks/Bonds (Public Company) **Kim Etchevers, MS**, Gilead Sciences, Inc.: Employee|Gilead Sciences, Inc.: Stocks/Bonds (Public Company) **Shuguang Chen, PhD**, Gilead Sciences, Inc.: Employee|Gilead Sciences, Inc.: Stocks/Bonds (Public Company) **Robert H. Hyland, DPhil**, Gilead Sciences, Inc.: Employee|Gilead Sciences, Inc.: Stocks/Bonds (Public Company) **Joe Llewellyn, PharmD**, Gilead Sciences, Inc.: Employee|Gilead Sciences, Inc.: Stocks/Bonds (Public Company) **Romas Geleziunas, PhD**, Gilead Sciences, Inc.: Employee|Gilead Sciences, Inc.: Stocks/Bonds (Public Company) **Antonella Castagna, MD**, Bristol-Myers Squibb: Advisor/Consultant|Gilead Sciences, Inc.: Advisor/Consultant|Gilead Sciences, Inc.: Grant/Research Support|Gilead Sciences, Inc.: Honoraria|Janssen: Advisor/Consultant|Janssen: Grant/Research Support|Janssen: Honoraria|Merck Sharp & Dohme: Advisor/Consultant|Merck Sharp & Dohme: Grant/Research Support|Merck Sharp & Dohme: Honoraria|ViiV Healthcare: Advisor/Consultant|ViiV Healthcare: Grant/Research Support|ViiV Healthcare: Honoraria **Charlotte Hedskog, PhD**, Gilead Sciences, Inc.: Employee|Gilead Sciences, Inc.: Stocks/Bonds (Public Company)

